# Application of a Novel Dissolution Medium with Lipids for In Vitro Simulation of the Postprandial Gastric Content

**DOI:** 10.3390/pharmaceutics16081040

**Published:** 2024-08-03

**Authors:** Tjaša Felicijan, Iva Rakoše, Manca Prislan, Igor Locatelli, Marija Bogataj, Jurij Trontelj

**Affiliations:** Department of Biopharmaceutics and Pharmacokinetics, Faculty of Pharmacy, University of Ljubljana, Aškerčeva cesta 7, 1000 Ljubljana, Slovenia; tjasa.felicijan@ffa.uni-lj.si (T.F.); iva.rakose@gmail.com (I.R.); manca.prislan@hotmail.com (M.P.); igor.locatelli@ffa.uni-lj.si (I.L.); jurij.trontelj@ffa.uni-lj.si (J.T.)

**Keywords:** fed-state medium, dissolution, SMOFlipid^®^, cinnarizine, HPLC analysis

## Abstract

Food can change various physiological parameters along the gastrointestinal tract, potentially impacting postprandial drug absorption. It is thus important to consider different in vivo conditions during in vitro studies. Therefore, a novel dissolution medium simulating variable postprandial pH values and lipid concentrations was developed and used in this study. Additionally, by establishing and validating a suitable analytical method, the effects of these parameters on the dissolution of a model drug, cinnarizine, and on its distribution between the lipid and aqueous phases of the medium were studied. Both parameters, pH value and lipid concentration, were shown to influence cinnarizine behavior in the in vitro dissolution studies. The amount of dissolved drug decreased with increasing pH due to cinnarizine’s decreasing solubility. At pH values 5 and 7, the higher concentration of lipids in the medium increased drug dissolution, and most of the dissolved drug was distributed in the lipid phase. In all media with a lower pH of 3, dissolution was fast and complete, with a significant amount of drug distributed in the lipid phase. These results are in accordance with the in vivo observed positive food effect on cinnarizine bioavailability described in the literature. The developed medium, with its ability to easily adjust the pH level and lipid concentration, thus offers a promising tool for assessing the effect of co-ingested food on the dissolution kinetics of poorly soluble drugs.

## 1. Introduction

The ingestion of food impacts several parameters along the highly variable and dynamic environment of the gastrointestinal (GI) tract. Administration of drugs in a postprandial state can influence drug dissolution, ionization, stability, or permeability indirectly by changing the conditions in the GI environment or directly by specific interactions [1]. Primarily, food ingestion impacts the conditions in the stomach, resulting in changes in gastric fluid volume, pH value, and motility pattern, as well as influencing gastric transit times [2,3,4,5]. In the distal parts of the GI tract, food’s effect on the characteristics of the fluids was less pronounced or insignificant; however, some changes could also be observed in the composition of the small and large intestine’s fluids, such as increased concentrations of bile acids and their salts, certain enzymes, or short-chain fatty acids [2,6,7].

For conducting the fed state bioequivalence and food interaction studies, EMA and the FDA recommend using a standardized high-fat meal [8,9]. A significant positive or negative food effect on drug bioavailability in a fed compared to the fasted state is demonstrated when the ratio of measured parameters (maximal plasma concentration (C_max_), the area under the plasma concentration–time curve (AUC)) is > 125% or < 80% (including the 90% confidence interval) [10]. An increase, decrease, acceleration, or delay in drug absorption can thus be observed due to food co-administration, which could influence drug efficacy and safety [11,12]. Among food components, lipids are often described as responsible for increasing the bioavailability of poorly soluble lipophilic drugs due to the formation of mixed micellar phases or vesicles that increase drug solubilization. Besides the lipid influence on drug dissolution, other mechanisms involving enhancing drug absorption may contribute to the increase in drug bioavailability [13,14].

One of the drugs that exhibits a food effect is cinnarizine (CIN), where the positive effect on the bioavailability of a conventional CIN tablet was demonstrated in an in vivo study on healthy volunteers as increased C_max_ and AUC [15]. CIN is a BCS class II weakly basic drug with two basic groups with pKa values of 1.95 and 7.47 and a log *p* value of 5.8 [16,17,18]. Besides being poorly water-soluble, it exhibits pH-dependent solubility, which was also reflected in the in vitro dissolution experiments [19]. Different solubilities within the range of physiological gastrointestinal pH values presumably produced high variability in the in vivo bioavailability studies [20,21]. Due to the pH-dependent solubility, intestinal precipitation can occur, which was correlated to variabilities in the observed C_max_, and one of the possible factors influencing the precipitation rate was bile salt concentration [22]. Formulating the drug as a self-nano-emulsifying drug delivery system (SNEDDS) showed increased bioavailability in the fasted state in dogs compared to the conventional tablet, and the positive food effect on CIN bioavailability was also minimized after application of the CIN SNEDDS formulation [23]. The inclusion of lipid components into formulation has thus been shown as a promising approach to overcome the poor solubility of CIN, and several researchers are studying the possibility and advantages of its inclusion in different lipid-based drug delivery systems [24,25,26,27,28]. Additionally, such systems could provide enhanced absorption through supersaturation generation due to the formation of mixed micelles and vesicles with absorbable lipids and the dilution of these lipid colloid phases by bile [29,30].

Due to the changes in the postprandial GI tract fluid composition and the possible food–drug interactions, it is essential to consider the conditions of the fed state in the in vitro dissolution studies. Milk or partially digested milk with pepsin has often been used in in vitro experiments as media to simulate gastric fluids in the fed state [13,31,32,33,34,35]. Other media frequently used in in vitro studies are nutritional drinks, such as Ensure^®^, which can also be used to establish the fed state in in vivo studies instead of a standardized meal [36,37]. Parenteral lipid emulsions, such as Lipofundin^®^ or Intralipid^®^, can also be used in dissolution studies to mimic the presence of lipids from food [38,39]. Additionally, due to changes in gastric content composition, three media based on milk or parenteral lipid emulsions were suggested in the literature to replicate the conditions in the stomach at various periods after meal ingestion [38,40]. Recently, another dissolution medium simulating different gastric conditions after ingesting a high-fat, high-calorie meal, FEDGAS, has also been commercially available at three different pH values [41]. FEDGAS has already been used in some dissolution studies to simulate postprandial gastric conditions [42,43,44]. 

Several media have thus already been described in the literature to simulate the in vivo fed-state gastric content. However, selecting a medium that enables variation in different parameters can be beneficial in assessing the influence of variable gastric conditions on drug dissolution. In our study, SMOFlipid^®^, a lipid injectable emulsion, was used in dissolution experiments and, according to our knowledge, has not yet been used for this purpose. SMOFlipid^®^ is composed of soybean oil, medium-chain triglycerides, olive oil, fish oil, egg phospholipids, glycerin, all-rac-alpha-tocopherol, sodium oleate, water, and sodium hydroxide [45].

Therefore, this work aimed to develop a dissolution medium simulating important parameters for poorly soluble drug dissolution, such as the lipid content and pH, which were varied within the physiological range of the postprandial stomach. A well-characterized lipid injectable emulsion was used as a source of lipids and surfactants in the medium. The independent control of selected parameters (lipid content and pH) was enabled within the developed medium by the proper dilution of lipid emulsion with buffers with different pH values. In addition to monitoring the dissolution of a model drug with a positive food effect, CIN, the drug’s partitioning into lipid and aqueous phases of the medium was also studied.

## 2. Materials and Methods

### 2.1. Materials 

Cinnarizine (CIN) tablets (Stugeron^®^, 75 mg, Janssen Pharmaceuticals, Beerse, Belgium) were purchased from a local pharmacy. The excipients in the tablets were the following: lactose monohydrate, corn starch, pregelatinized starch, povidone, polysorbate 20, microcrystalline cellulose, anhydrous colloidal silica, and magnesium stearate [46]. SMOFlipid^®^ (Fresenius Kabi, Bad Homburg vor der Höhe, Germany) was purchased from a local pharmacy. Disodium hydrogen phosphate (Na_2_HPO_4_), citric acid monohydrate (C_6_H_8_O_7_∙H_2_O), and formic acid (FA) (98–100%) were purchased from Merck KGaA (Darmstadt, Germany). CIN powder (>99%) and HPLC analytical grade acetonitrile (AcN) (>99.9%) were purchased from Sigma Aldrich (Steinheim, Germany) and Honeywell (Seelze, Germany), respectively. Ultra-pure water was prepared using the Purelab classic ultrafiltration apparatus (ELGA Lab-water, High Wycombe, UK).

### 2.2. Media Preparation

Different volumetric ratios of 0.1 M citric acid and 0.2 M disodium hydrogen phosphate solutions were mixed and suitably diluted using purified water to obtain 4-times diluted McIlvaine buffers (DMBs) of pH values 3, 5, and 7. pH was measured using the Mettler Toledo Seven Compact pH meter (Mettler Toledo, Switzerland) and adjusted with citric acid or disodium hydrogen phosphate solutions to achieve the desired pH value ± 0.05 unit. DMBs were used in our study as a buffer system because of their capability to maintain sufficient buffer capacity over a wide range of pH values. Pure DMBs were used in dissolution experiments as media that do not contain lipids and simulate fed gastric conditions only with pH. SMOFlipid^®^, a well-characterized and nutritionally relevant source of lipids [45,47,48], was used in the media to mimic the lipid food components. SMOFlipid^®^ with a 200 mg/mL lipid concentration was diluted with DMBs to obtain media with lipid concentrations of 8.8 mg/mL and 35.2 mg/mL for dissolution studies and 8.8 mg/mL and 100 mg/mL for the mass balance confirmation experiments. No phase separation was visible after the dilution of SMOFlipid^®^ emulsion with DMBs, and it was thus assumed that the prepared medium was homogeneous. The pH value of the final medium was equal to the pH value of pure DMB regardless of the lipid concentration.

### 2.3. Sample Preparation

The samples had to be suitably processed before performing the HPLC analysis to determine the CIN concentration. The procedures differed considering the presence of lipids in the medium and are presented in the following subsections.

#### 2.3.1. Media without Lipids

In experiments where only DMBs (without lipids) were used as media, samples were diluted suitably before HPLC analysis with a dilution solvent that consisted of equal volumetric parts of 0.2% FA and AcN.

#### 2.3.2. Media with Lipids

**Figure 1:**
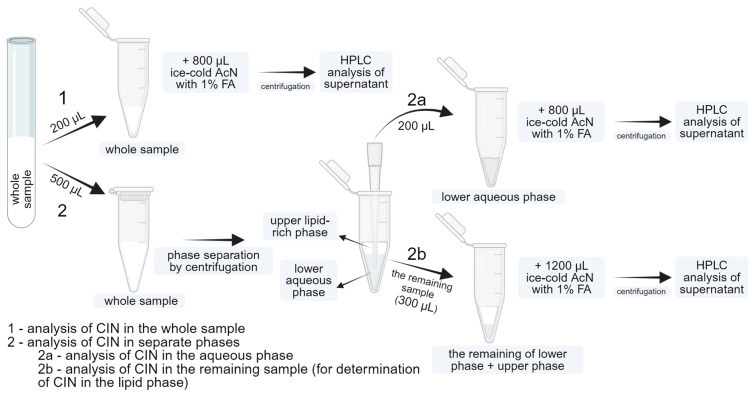
A scheme of sample preparation procedures in media with lipids for the determination of cinnarizine (CIN) in the whole sample and the separate aqueous and lipid phases.

A scheme of sample treatments before analysis of the amount of CIN in the whole sample and different phases in media containing lipids is presented in Figure 1 and described in the following sections.

*a*. *Sample preparation for determining the amount of CIN in the whole sample*

The total amount of dissolved CIN (procedure 1 in Figure 1) was determined by mixing 200 µL of the whole sample (i.e., untreated sample without the preceding phase separation) with 800 µL of ice-cold AcN containing 1% (*v*/*v*%) FA, vortex-mixed for 10 s and centrifuged for 10 min at 21,300× *g* at 5 °C (Centrifuge 5425 R, Eppendorf, Hamburg, Germany). A 500 µL aliquot of the supernatant was transferred to a vial and analyzed using the HPLC method.

*b*. *Sample preparation for determining the amount of CIN in the separate phases*

To determine the amount of CIN in the lipid and aqueous phases of the sample (procedure 2 in Figure 1), phase separation was first performed by centrifugation of 500 µL of the whole sample at 20 °C for 20 min and 21,300× *g*. After centrifugation, two phases were visible: the lower aqueous phase and the upper lipid-rich phase, which was distinguished from the transparent aqueous phase by its white color. A total of 200 µL of the lower aqueous phase was withdrawn and mixed with 800 µL of ice-cold AcN containing 1% FA, vortex mixed for 10 s, and centrifuged again for 10 min at 21,300× *g* at 5 °C. The remaining sample in the microcentrifuge tube from the first centrifugation (300 μL) was also diluted 5 times using ice-cold AcN containing 1% FA and treated the same way as the lower phase. The supernatants were analyzed by HPLC. A few assumptions had to be adopted to calculate the amount of CIN in separate phases of the sample, such as the theoretical volumes of each phase and the density of the lipid phase. A detailed description of assumptions used in calculations is given in the Supplementary Materials.

### 2.4. HPLC Analysis

The samples were analyzed on an Agilent 1100/1200 series liquid chromatograph (Agilent Technologies, Santa Clara, CA, USA) equipped with a quaternary pump and a variable wavelength UV detector set at 251 nm. The mobile phase A consisted of 0.2% FA in ultra-pure water, and the mobile phase B was 99.9% acetonitrile with 0.1% ultra-pure water. The chromatographic separation of a sample with an injection volume of 5 µL was performed on a Gemini NX C18 50 × 3 mm, 3 µm column with a Phenomenex C18, 4 × 3 mm guard column (Phenomenex, Torrance, CA, USA) maintained at 45 °C. The flow rate of the mobile phase was set to 1.5 mL/min, and the analyte was eluted by gradient elution from 20% B to 65% B over 1.5 min, then maintained at 65% for 2 min, and then lowered back to 20% B. The total analysis run-time, including re-equilibration, was 5 min.

#### HPLC Method Validation

The HPLC method has been validated based on the new draft ICH guidelines [49] for method validation for the following parameters: selectivity, linearity and working range, precision, accuracy, recovery, analyte stability, and robustness.

*a*. *Selectivity*

**Figure 2:**
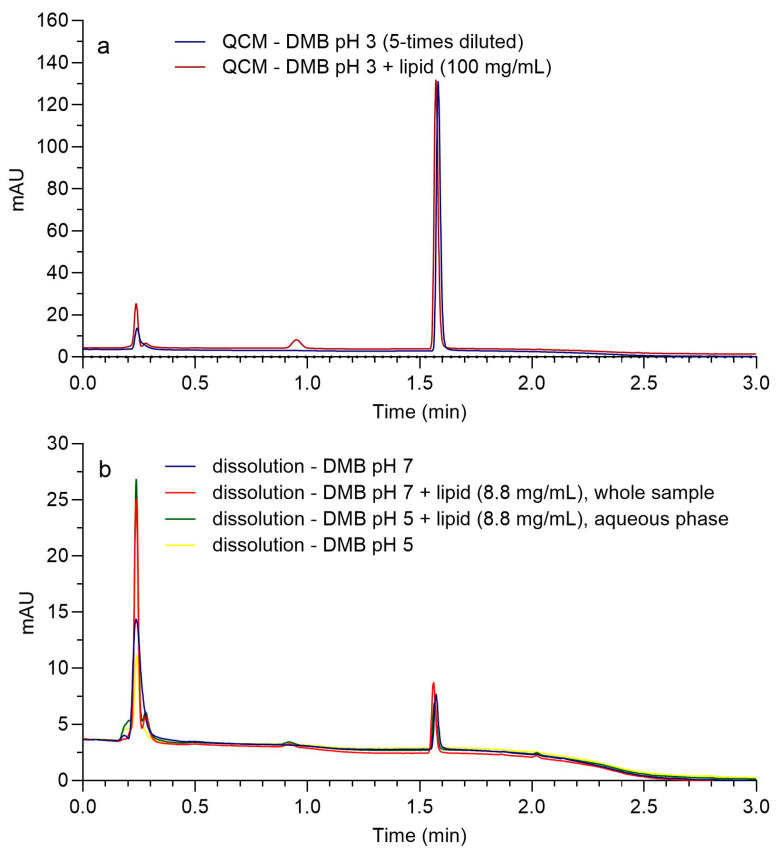
HPLC chromatograms of different samples in the experiments: (**a**) QCM standards in DMB pH 3 without lipids, suitably diluted with solvent to match the concentration of QCM in media with lipids, and in DMB pH 3 with 100 mg/mL of lipids; (**b**) chromatograms of samples from dissolution experiments with a response near the LLOQ in media with and without lipids at pH values 5 or 7. The numerical data of each chromatogram were exported from Agilent ChemStation software (version B.04.03) and redrawn using GraphPad Prism.

The absence of chromatographic interference from the formulation or dissolution medium demonstrated the method’s selectivity. Figure 2a shows chromatograms of cinnarizine standard (medium-level quality control with CIN concentration around 75 mg/L, QCM) in media with and without lipids at pH 3, and Figure 2b shows sample chromatograms from the dissolution experiments in media with and without lipids at pH values 5 and 7 at response levels in the range of the lower limit of quantification (LLOQ, CIN concentration around 0.5 mg/L).

*b*. *Linearity and working range*

The linearity and working range were evaluated by plotting the method response (peak area) against the spiked analyte concentration in different media used for calibration: for media without lipids in pure dilution solvent and for media with lipids in 0.2% FA, which was further processed the same way as the samples in experiments, i.e., diluted 5 times using the ice-cold AcN containing 1% FA and centrifuged, as described above in Section 2.3. Two calibration curves were prepared in each medium to adapt to the varying solubility ranges of CIN in different media. The results are presented in Table 1. The linear relationship was determined by visual inspection of the data points’ distribution, and the linear regression analysis was performed to obtain the calibration parameters and the determination coefficient (R^2^), which should be higher or equal to 0.999. The lower limit of quantitation was established as the lowest calibration sample, demonstrating the required accuracy (90–110%), signal-to-noise (>10:1), and signal-to-blank ratios (>10:1).

*c*. *Accuracy and recovery*

The accuracy was determined as the mean percent recovery of the spiked analyte into the dissolution medium. The recovery was calculated as a quotient of found and expected concentrations by Equation (1), where $c_{found}$ represents the measured concentration after spiking and $c_{expected}$ represents the theoretically expected concentration.

(1) $$recovery=\frac{c_{found}}{c_{expected}}\times 100\%$$

The spiking was performed at least in three replicates of quality control (QC) samples at each of the four concentration levels, i.e., at two low concentrations (QCL-1 and QCL-2, with CIN concentrations around 10 and 35 mg/L, respectively), a medium concentration around 75 mg/L (QCM), and a high standard concentration around 115 mg/L (QCH). The average measured concentration at each level was compared to the true spiked concentration with acceptance criteria of 95–105% and 85–115% for dissolution media containing only buffer solutions and media containing lipids, respectively. The accuracy data are presented in Table 2.

*d*. *Precision*

Within-run precision was determined by comparing the analyte response obtained from at least three separately prepared and analyzed parallels of the same QC sample at four concentration levels (QCL-1, QCL-2, QCM, and QCH). The between-run precision was evaluated by comparing the analyte response from aliquots of the same QC sample obtained on three different days. The precision was deemed acceptable if RSD was below 5% and 15% for media containing only buffer solutions and media with added lipids, respectively. The results are presented in Table 3.

*e*. *Analyte stability and method robustness*

The analyte’s stability was tested in different dissolution media before and after sample preparation. The method’s robustness was tested by analyzing the QC samples with different representative CIN concentrations before and after performing small, deliberate changes in chromatographic parameters to simulate the maximal possible variation during its use. The results are given in the Supplementary Materials (Tables S1a–g and S2a–c).

### 2.5. Mass Balance Confirmation

The mass balance was evaluated in separate experiments by spiking the blank media sample with the standard at each of the three QC levels (QCL-2, QCM, and QCH, with concentrations around 35, 75, and 115 mg/L, respectively). Media with different pH values (3, 5, and 7) and two lipid concentrations (8.8 mg/mL and 100 mg/mL) were used. For QCL-2 and QCH levels, the mass balance was determined at three time points, i.e., immediately after standard addition to the medium (0 h) and after 4 and 24 h, during which the spiked samples were constantly agitated at room temperature. The experiments were performed in triplicate. The mass balance for QCM and the amount of CIN in the whole sample were determined at the 0 h time point. The analyte concentration was determined in 200 µL of the whole sample (as seen in Figure 1). The CIN mass was then determined separately in the aqueous and lipid phases of the 500 µL sample according to the procedure described in Section 2.3, and the percentage of CIN in each phase was calculated considering the determined CIN mass in the whole sample. The sum of CIN percentages from both phases was then compared to the theoretical 100% and used to evaluate the mass balance and consistency of analyte recovery from both phases. The mass balance was also estimated during dissolution studies to confirm the complete recovery and accuracy of the method in the actual dissolution samples.

### 2.6. Dissolution Experiments

Dissolution experiments were performed in 500 mL of medium using a paddle apparatus (Agilent 708-DS, Agilent Technologies, Santa Clara, CA, USA) at 37 ± 0.5 °C and a paddle stirring speed of 75 rpm. Tablets were inserted into wire sinkers to prevent them from sticking to the bottom of the vessel. Sampling was performed automatically through 10 µm UHMWPE full-flow filters (Agilent Technologies, Santa Clara, CA, USA). During the experiments, the volume of the medium used in sampling (3 mL) was not replaced, but the decrease in volume and loss of drug due to sampling were included in the calculations. Samples were prepared according to the procedures described in Section 2.3. The experiments were performed in triplicate.

### 2.7. Data Analysis

The calculations were performed using Microsoft Excel 2019 (Microsoft Corporation, Redmond, DC, USA), while explanatory and statistical data analysis was performed in the GraphPad Prism 10.1.0 software (GraphPad, Boston, MA, USA). A one-way analysis of variance (ANOVA) with equal variances and the Bonferroni post hoc test were used to statistically analyze the results. The effect of pH value on CIN distribution into the lipid phase was statistically evaluated from mass balance and dissolution experiments. Namely, for the mass balance data, the effect of pH was compared as a difference between groups with the same pH value (i.e., including all 21 determinations of CIN in the lipid phase; 7 experiments by 3 aliquots) separately for low and high lipid concentrations. In dissolution experiments, the effect of pH was evaluated at the 120 min time point (i.e., the last time point measured at all pH values) separately for low and high lipid concentrations as well. A mean difference with a 95% confidence interval (CI) was reported, with a threshold of *p* < 0.05 indicating statistical significance.

## 3. Results and Discussion

One of the main challenges in implementing a versatile biorelevant dissolution medium is the high intra- and inter-individual variability of the GI tract fluids, exhibited already in the fasted state and further significantly affected by food ingestion [12,50]. Medium pH value and lipid concentration are two essential, independent, and highly variable parameters that could significantly affect drug dissolution and were thus selected to be simulated in our experiments. After a high-fat meal, the amount of fat in the stomach increases significantly and can present up to approximately 10% of the total gastric content, as measured by the MRI, which then decreases over time [51]. Similarly, elevated gastric pH values (around pH 4.6) are found postprandially and then gradually decrease back to a basal pH value, but the pH is highly variable and can increase even above pH 6 [52,53,54]. Lipofundin^®^, a parenteral lipid emulsion [55], was suggested to be used as a source of lipids in dissolution media [38] to simulate the lipid concentration in the stomach after a meal as previously measured in vivo [56,57]. A later study [58] concluded that the proposed late level II fed-state simulated gastric fluid with Lipofundin^®^ [38] reflects well the total molar concentration of lipid species and osmolality in an average fed state stomach content. In our study, SMOFlipid^®^, a lipid injectable emulsion containing 200 mg/mL lipids deriving from a mixture of soybean oil, medium-chain triglycerides, olive oil, and fish oil [45], was used. A detailed composition of the used emulsion is given in the Supplementary Materials. There are some differences in the composition of both emulsions (SMOFlipid^®^ and Lipofundin^®^), but the lipid concentration was the same in both emulsions, and data about the medium containing Lipofundin^®^ from the literature were used as a foundation for developing our medium. The ratios between Lipofundin^®^ and buffer, as suggested in the literature, were 17.5:82.5, 8.75:91.25, and 4.375:95.625 for early, middle, and late simulated fed state gastric medium, respectively [38]. In our study, the two extreme dilutions of the lipid emulsion were used, and SMOFlipid^®^ was diluted with DMBs with a pH value of 3, 5, or 7. After calculating the dilution factor and considering the lipid content in the emulsion, the concentrations of lipids in the tested media in the dissolution studies were 35.2 mg/mL and 8.8 mg/mL, which is in accordance with lipid concentrations in early and late fed-state simulated gastric fluids proposed in the literature using Lipofundin^®^ [38].

### 3.1. HPLC Method Validation

The presence of lipids in media could generate several analytical challenges. Thus, one of the aims of this study was to develop a suitable sample preparation procedure and HPLC method for CIN determination in media containing different concentrations of lipids. As can be seen from HPLC chromatograms in Figure 2a, the presence of lipids in the medium did not interfere with the CIN peak, and the responses of analyte standard (QCM) in medium without and with lipids (concentration of 100 mg/mL) at pH 3 were similar. Additionally, no interference of the formulation excipients with the CIN peak was observed, as seen in Figure 2b, where the HPLC chromatograms from dissolution experiments at pH values 5 and 7 without and with lipids with analyte peak responses near LLOQ (peak response around 5 mAUs) are presented. The selectivity of the method was thus confirmed.

To calculate the analyte concentration, calibration curves were prepared in two media: in solvent for samples without lipids and in 0.2% FA using the procedure with ice-cold AcN containing 1% FA for samples with lipids. The results are presented in Table 1. Due to the wide range of concentrations in the experiments, two calibration curves were prepared in each medium, one covering the normal range of expected concentrations and the other specifically covering the lower concentration range. Some calibration standards were prepared separately for both calibrated ranges, and their calculated concentrations were acceptably accurate using both calibration line equations. All calibration curves were linear (assessed by visual inspection) with R^2^ > 0.999.

The suitability of the prepared calibration curves for calculating the amount of CIN in samples with different compositions was evaluated by preparing QC samples in all tested media. The accuracy and precision were within the pre-set limits, as seen in Table 2 and Table 3. Additionally, the analyte extraction from the lipid matrix requires a particular sample preparation, which can influence the recovery efficiency. This was observed in a similar study, where they developed the HPLC method to determine caffeine in two media containing lipids: milk and a lipid emulsion, Intralipid^®^ [59]. Ice-cold 12% trichloroacetic acid was used for emulsion separation or protein precipitation, and the recovery efficiency from lipid emulsion was noticeably lower than that of milk media. However, the extraction procedure using ice-cold acetonitrile with formic acid resulted in a repeatably complete recovery in our study (Table 2 and Table 3). Based on these results, the method’s suitability was confirmed.

### 3.2. Mass Balance Confirmation

**Figure 3:**
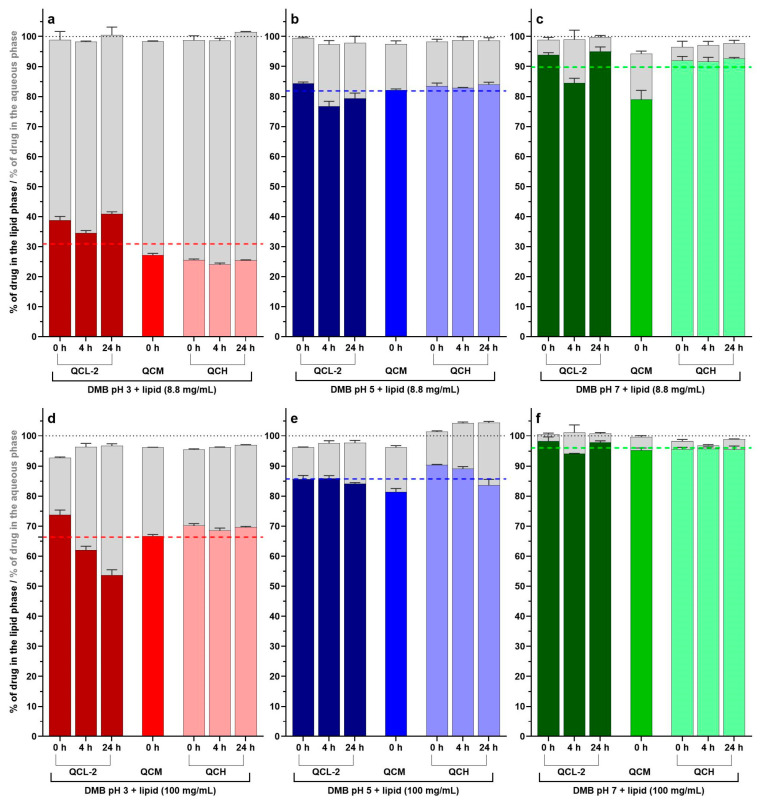
The percentage of cinnarizine in the lipid (color bars) and aqueous (gray bars) phases of QC standards in media with pH 3 (**a**,**d**), 5 (**b**,**e**), and 7 (**c**,**f**) and at lipid concentrations of 8.8 mg/mL (**a**–**c**) and 100 mg/mL (**d**–**f**). The average results of three replicates with standard deviations are presented for each of the three drug concentration levels (i.e., low, QCL-2, medium, QCM, and high, QCH) at different time points after preparation (0, 4, or 24 h). The dashed colored lines represent the mean of 21 determined percentages of cinnarizine in the lipid phase, and a black dotted line represents the 100% axis mark.

The measured concentration of CIN in the whole sample and the percentage of CIN in the aqueous and lipid phases for all measured time points in mass balance experiments are presented in Figure 3. The percentage of CIN in each phase was calculated in relation to the CIN determined in the whole sample. The numerical data are given in Supplementary Materials in Table S3a,b.

The measured CIN concentrations in the whole sample deviated from the theoretical concentrations of CIN added to the sample by less than ±10% in all media tested, supporting the suitability of our analytical method. Additionally, the sum of determined percentages of CIN in the lipid and aqueous phases was between 93% and 105% for all media (Table S3a,b in the Supplementary Materials). This result further confirms the applicability of the developed method for analyzing samples with different compositions and the reliability of CIN distribution results, despite the use of some assumptions in the calculations.

The distribution of CIN between aqueous and lipid phases depends on several parameters, as seen in Figure 3. The percentage of CIN in the lipid phase statistically significantly increased with increasing pH values (ANOVA test, *p* < 0.001) due to the decreasing percentage of protonated CIN and, thus, the lower aqueous solubility of the drug at higher pH values. This was observed in both lipid concentrations, namely 8.8 and 100 mg/mL. Post hoc pairwise analysis revealed that the mean difference in the percentage of CIN in the lipid phase between pH values 5 and 3 was 51.0% (95% CI = 46.9–55.0%, *p* < 0.001, 42 measurements) at the lower lipid concentration of 8.8 mg/mL and 19.4% (95% CI = 16.2–22.6%, *p* < 0.001, 42 measurements) at a lipid concentration of 100 mg/mL. At pH value 7, almost all CIN was located in the lipid phase. However, the difference in the percentage of CIN in the lipid phase between pH values 7 and 5 was also statistically significant, with mean differences of 7.9% (95% CI = 3.8–12.0%, *p* < 0.001, 42 measurements) and 10.3% (95% CI = 7.1–13.5%, *p* < 0.001, 42 measurements) at lower and higher lipid concentrations, respectively. Nevertheless, at pH 3, where CIN exhibits good solubility in the aqueous phase, a high amount of CIN was still distributed in the lipid phase. This is in accordance with literature data from the CIN partitioning experiment, where a large amount of CIN was found in the organic phase at low pH values of the aqueous phase between 1.0 and 3.0 [19].

To assess the kinetics of CIN distribution into the lipid phase, measurements of CIN in the separate phases were performed at different time points after preparation of standards for mass balance confirmation experiments, i.e., immediately after preparation (t = 0 h), after 4 h, and after 24 h. In most media, minor differences in the distribution between phases at different time points were observed. Considering the data for CIN solubility in soy oil (27.6 mg/g) found in the literature [60], it was assumed that the solubility of CIN in the lipid phase was not achieved in any media, not even at the highest CIN standard (QCH) at pH 7 with the lowest amount of lipids (8.8 mg/mL), where the determined amount of CIN did not exceed 14 mg per gram of lipid. However, as SMOFlipid^®^ also contains other lipids, not only soy oil, this number is only an estimation of CIN solubility in the lipid components of our media. It was additionally reported that the solubilization capacity may depend on the type of colloidal structures present, as seen in a study where different CIN solubilities were observed in media with two specific ratios between oleic acid and monooleate that exhibited different nanostructures–micelles or vesicles [17]. Nevertheless, the mass balance results at different times indicate a fast distribution of CIN into the lipid phase, even in cases of higher CIN concentrations in the lipid.

Furthermore, at the 24 h time points in experiments with QCL-2 standard in medium DMB pH 3 + lipid (100 mg/mL) and QCH standard in DMB pH 5 + lipid (100 mg/mL), separated lipid droplets were observed in the upper phase of the sample after phase separation, which was not seen at other CIN concentrations in the same media nor in mass balance experiments at any other condition. In the literature, the stability of the emulsions loaded with the drug was described as being influenced by their charge [61]. The lipid droplets seen in our experiment could indicate emulsion instability due to the presence of the drug, which could influence the distribution of CIN in the lipid phase at the 24 h time point measurement, as observed in Figure 3.

### 3.3. The Influence of Medium pH Value and Lipid Concentration on CIN Release

**Figure 4:**
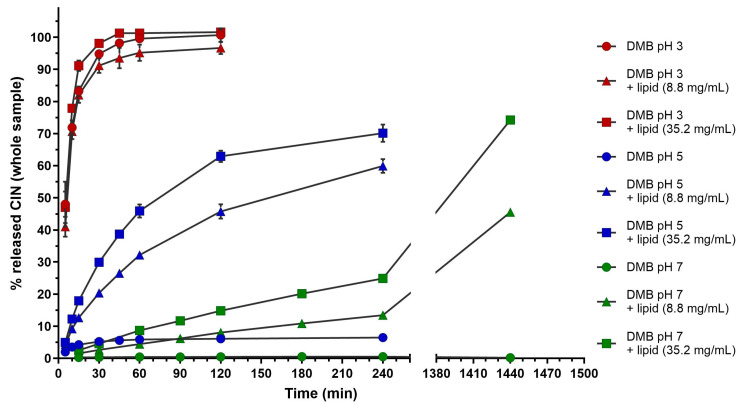
The percentage of released cinnarizine, determined in whole samples in dissolution studies using media with different pH values and lipid content. Average values of three parallels with standard deviations are presented, except for the 1440 min time point, where only one parallel was performed.

The results of separate mass balance confirmation experiments illustrated the tendency for CIN distribution between the lipid and aqueous phases in media with different lipid content and at different CIN concentrations. However, a pre-dissolved CIN standard in an organic solvent was added to the medium in these experiments. In dissolution experiments, other factors could also influence CIN behavior in media containing lipids, such as drug dissolution, its release from the formulation, and the presence of different excipients in the dosage form, which could influence CIN solubility and its distribution between the aqueous and lipid phases. Dissolution experiments were thus performed with immediate-release CIN tablets using the developed media containing lipids. The amount of dissolved CIN, determined in the whole sample without preceding phase separation, was first measured in dissolution studies to assess the effect of pH value and lipid content on drug release. The results for all media are presented in Figure 4.

As seen in Figure 4, the medium pH value greatly influenced CIN dissolution. According to the drug’s pKa values of 1.95 and 7.47 [16,18], the CIN release was immediate and complete at pH 3, and in other media, the amount of released CIN decreased with increasing pH. Similar differences in dissolution rate in buffers at pH values 3 and 5 were already observed in the literature [19]. These results are also in accordance with literature data on CIN solubility, which was approximately 16 times lower at pH 6.9 compared to pH 5 and approximately 140 times lower at pH value 5 than at pH 2.5 [19].

The pH value also influenced CIN release in media with lipids. As CIN exhibited a fast release at pH 3, the addition of lipids at either concentration (8.8 and 35.2 mg/mL) did not further accelerate its release. On the contrary, adding lipids to the dissolution medium at higher pH values (5 and 7) increased CIN release compared to dissolution in pure DMBs. The effect of added lipids was especially observed at pH 5, but CIN release increased at pH 7, where the solubility was the lowest. At this pH value, the amount of CIN was also measured after 24 h in one parallel, and approximately 74% of CIN (concentration around 115 mg/L) was released in an experiment with the highest lipid concentration in the medium (35.2 mg/mL). At pH values 5 and 7, the concentration of lipids was also an important factor for CIN dissolution, with a higher lipid content causing a faster/greater CIN release. However, the release at pH values 5 and 7 was lower than at pH 3, despite the addition of lipids. Presumably, the solubility in the aqueous phase influences CIN release, and due to its limited solubility at pH 5 and 7, the effect of lipids could not prevail over the effect of pH value. Therefore, the effect of lipids could be more pronounced with lower CIN dosages, i.e., 25 mg, as used in the in vivo study [15], where the positive food effect was observed.

### 3.4. The Influence of Medium pH Value and Lipid Concentration on CIN Distribution between Aqueous and Lipid Phases in Dissolution Experiments

**Figure 5:**
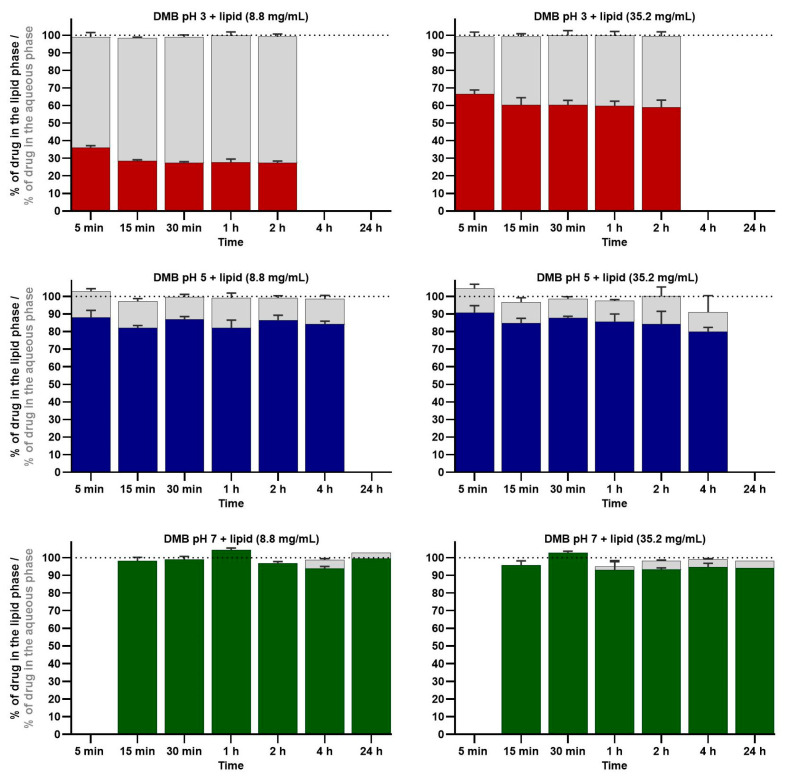
The percentage of cinnarizine in the lipid (color bars) and aqueous (gray bars) phases during dissolution studies in different media with lipids. The percentages were calculated in relation to the concentration of CIN in the whole sample at corresponding time points. The experiments were performed in triplicate (except for 24 h time points, where only one experiment was performed). Averages and standard deviations are presented, and the black dotted line represents the 100% axis mark.

The distribution of released CIN into aqueous and lipid phases, calculated as the percentage of dissolved CIN in each phase in relation to the measured dissolved CIN in the whole sample, was also determined in each sample in the dissolution experiment. Similar to the mass balance results, the sum of CIN percentages in the lipid and aqueous phases approached 100%. This result indicates that the analytical method of determining CIN in the separate phases was also applicable to samples from the dissolution experiments, which is important since it was already reported in the literature that the excipients from the tablet could influence CIN extraction efficiency [62]. The average results of CIN distribution between both phases in dissolution experiments are presented in Figure 5.

The effect of medium pH value and lipid concentration resulted in similar trends of CIN distribution into each phase in dissolution experiments than observed previously in the mass balance confirmation experiments. The results of the ANOVA test for the 2 h time point of the dissolution experiments showed a statistically significant influence of the medium pH value in both lipid concentrations (*p* < 0.001). Furthermore, the post hoc pairwise analyses between pH values 5 and 3 revealed statistically significant mean differences in the percentage of CIN in the lipid phase, namely 59.0% (95% CI = 54.0–64.0%, *p* < 0.001) at a lower lipid concentration of 8.8 mg/mL and 25.3% (95% CI = 12.1–38.4%, *p* = 0.002) at the higher lipid concentration of 35.2 mg/mL. Similar to the mass balance results, the differences in the amount of CIN in the lipid phase in media with higher pH values 7 and 5 were less pronounced but were still statistically significant at a lipid concentration of 8.8 mg/mL with a mean difference of 10.5% (95% CI = 5.5–15.5%, *p* = 0.001). At the higher lipid concentration of 35.2 mg/mL, the mean difference was 9.2% (95% CI = −4.0–22.4%) but was not statistically significant (*p* = 0.187) due to the higher variability in results at pH value 5.

As seen in Figure 5, the lipid concentration in dissolution media did not influence CIN distribution into the lipid phase at the highest pH values (5 and 7). On the contrary, the lipid concentration at pH 3 notably influenced the CIN distribution between phases in the dissolution experiments, where the percentage of CIN in the lipid phase was higher when a dissolution medium with a higher lipid concentration was used. During the dissolution studies, the amount of dissolved CIN in the whole sample increased with time, but the percentage of CIN distributed into the lipid phase slightly decreased. Presumably, this could occur due to the coexistence of several equilibria, potentially influencing drug dissolution and distribution kinetics.

At the beginning of the experiment, CIN is released from the tablet in the aqueous phase of the medium, where an equilibrium between ionized and un-ionized CIN is established, depending on the medium pH value and according to the drug’s pKa. Afterward, the distribution of CIN into the lipid phase occurs, further facilitating drug dissolution in the aqueous phase. A high percentage of CIN distributed into the lipid phase at pH 3 shows a high affinity of CIN for the lipid-rich phase even when ionized and reflects good solubility of CIN in the lipids, which was also observed previously in the literature [63]. However, since the mass balance results show a fast distribution of the dissolved CIN into the lipid phase, we presume that the rate-limiting step governing CIN behavior in media containing lipids is its release from the tablet in the aqueous phase. The obtained dissolution results are thus influenced by all the main processes mentioned above: drug dissolution and ionization in the aqueous phase, as well as CIN distribution. Considering the lipid content and pH ranges of the newly developed dissolution medium are close to the reported in vivo fed conditions in the stomach, such a system provides valuable insight into drug behavior in the postprandial state. Its main benefit is the possibility of selectively simulating some of the most important conditions for the dissolution of poorly soluble drugs. However, the in vivo situation with many additional processes, such as bile secretion, the formation of micelles and vesicles, and the presence of other luminal components and movements, is far more complex than the described novel in vitro dissolution system. Nevertheless, its simplicity and straightforward analytics offer repeatable and in vivo-representable results, which can provide important insight into drug behavior in the postprandial stomach.

## 4. Conclusions

This work focused on implementing a novel dissolution medium in the in vitro CIN release study. Several in vivo variables of the postprandial gastric fluids were simulated in a simple in vitro dissolution system with a newly developed dissolution medium, considering variable lipid concentrations and pH values. A robust analytical method was established and validated to determine the amount of dissolved drug in the samples with different lipid concentrations. Additionally, by analyzing the drug amount in the separate phases of the sample, the mass balance was confirmed, and the CIN’s tendency to distribute between the lipid and aqueous phases could be evaluated. It was shown that the release of a weakly basic model drug was strongly dependent on pH, i.e., its release was higher at lower pH, and the release also increased with an increasing amount of added lipid at pH values where the solubility was low. This is in accordance with the positive food effect observed in several in vivo studies described in the literature. Thus, the obtained results demonstrate the suitability of the developed medium to assess the behavior of drugs in the simulated fed conditions, especially for drugs that express low and pH-dependent solubility and are well soluble in lipids. Additionally, the easy modification of the medium, especially its composition, provides further possibilities for its optimization and implementation in dissolution studies.

## Figures and Tables

**Table 1:** The parameters of the calibration curves used to determine the amount of drug in different media.

Calibration Curve				
	For Media without Lipids		For Media with Lipids	
	Solvent (Lower Range)	Solvent (Normal Range)	0.2% FA (Lower Range)	0.2% FA (Normal Range)
concentration range (mg/L)	0.51–2.56	1.76–88.02	0.55–13.12	6.75–168.75
slope	10.596	10.389	2.142	2.105
intercept	−0.125	−1.4118	−0.140	−1.150
R^2^	0.9999	1.0000	0.9995	1.0000

**Table 2:** Accuracy of the analytical method in media with different pH values and lipid concentrations. Three parallels were performed in each medium, except in DMB pH 3 without lipids (at all QC levels) and in DMB pH 5 without lipids at QCL-1, where 6 parallels were performed. N.D.—not determined.

Accuracy (%)										
	SMOFlipid^®^ (Undiluted)	DMB pH 3			DMB pH 5			DMB pH 7		
		No Lipid	+Lipid 8.8 mg/mL	+Lipid 35.2 mg/mL	No Lipid	+Lipid 8.8 mg/mL	+Lipid 35.2 mg/mL	No Lipid	+Lipid 8.8 mg/mL	+Lipid 35.2 mg/mL
QCL-1	115.0	97.5	99.0	103.3	96.6	109.1	105.3	N.D.	101.9	110.0
QCL-2	107.1	96.9	97.5	101.8	95.0	102.9	104.9	N.D.	97.6	101.2
QCM	106.8	98.8	94.8	97.2	N.D.	100.3	103.4	N.D.	96.8	102.7
QCH	105.2	100.8	101.6	99.4	N.D.	96.8	104.3	N.D.	96.4	97.7

**Table 3:** The precision data for the analytical method in media with different pH values and lipid concentrations. Three parallels were performed in each medium, except in DMB pH 3 without lipids (at all QC levels) and in DMB pH 5 without lipids at QCL-1, where 6 parallels were performed. For QCL-1 in DMB pH 7 without lipids, 9 parallels were performed. N.D.—not determined.

Precision (% RSD)										
	SMOFlipid^®^ (Undiluted)	DMB pH 3			DMB pH 5			DMB pH 7		
		No Lipid *	+Lipid 8.8 mg/mL	+Lipid 35.2 mg/mL	No Lipid *	+Lipid 8.8 mg/mL	+Lipid 35.2 mg/mL	No Lipid *	+Lipid 8.8 mg/mL	+Lipid 35.2 mg/mL
QCL-1	2.4	1.4	3.3	3.4	3.1	2.3	2.1	21.8 **	5.1	3.1
QCL-2	2.5	2.3	0.8	1.9	1.0	2.6	0.5	N.D.	2.0	0.4
QCM	2.9	3.1	3.4	0.9	N.D.	1.5	1.1	N.D.	2.4	1.4
QCH	3.4	1.2	0.5	0.8	N.D.	4.0	2.7	N.D.	2.2	0.3

* between-day precision. ** Due to limited solubility, the precision was outside of the defined acceptance criteria.

## Data Availability

The raw data supporting the conclusions of this article will be made available by the authors upon request.

## Supplementary Materials

The following supporting information can be downloaded at: https://www.mdpi.com/article/10.3390/pharmaceutics16081040/s1, 1. The composition of SMOFlipid^®^; 2. Assumptions used in calculations to determine the amount of CIN in the separate phases [64]; 3. HPLC method validation—analyte stability and method robustness; Table S1a: Testing the changes in the column temperature (±5 °C) on analyte response; Table S1b: Testing the changes in the detector wavelength (± 2 nm) on analyte response; Table S1c: Testing the changes in mobile phase flow rate (± 0.15 mL/min) on analyte response. A ratio between the tested and reference mobile phase flow rates was used to calculate the expected concentration at the tested flow rates; Table S1d: Testing the changes in the initial percentage of the mobile phase A (± 5%) on analyte response; Table S1e: Testing the changes in mobile phase A pH value (± 0.3 units) on analyte response; Table S1f: Testing the changes in injection volume (± 1 µL) on analyte response. A ratio between tested and reference injection volumes was used to calculate the expected concentration at tested injection volumes; Table S1g: Automatic and manual integration of seven quality QC standards with nominal CIN concentrations between approximately 7 and 175 mg/L. Manual integration was performed individually by two analysts; Table S2a: The analyte stability before sample preparation in medium DMB pH 3 + lipid (8.8 mg/mL). The results of two or three parallels are presented for all three time points. The concentrations obtained at 0 h time point are used as a reference for accuracy calculations at other time points. The precision is calculated as % RSD from all measurements. N.D.—not determined; Table S2b: The analyte stability before sample preparation in medium DMB pH 3 + lipid (100 mg/mL). The results of two or three parallels are presented for all time points. The concentrations obtained at 0 h time point are used as a reference for accuracy calculations at other time points. The precision is calculated as % RSD from all measurements. N.D.—not determined; Table S2c: The analyte stability after sample preparation in medium DMB pH 3 + lipid (100 mg/mL). The results are presented for two time points; 4. Mass balance confirmation experiments; Table S3a: Mass balance results in media with a lipid concentration of 8.8 mg/mL at three pH values. The average determined concentration (mg/L) of CIN in the whole sample and the average percentage of CIN in the aqueous and lipid phases of three parallels at different time points after standard preparation (0 h, 4 h, 24 h) are presented. Standard deviations (SD) are presented in brackets. N.D.—not determined, 95% CI–95% confidence interval; Table S3b: Mass balance results in media with a lipid concentration of 100 mg/mL at three pH values. The average determined concentration (mg/L) of CIN in the whole sample and the average percentage of CIN in the aqueous and lipid phases of three parallels at different time points after standard preparation (0 h, 4 h, and 24 h) are presented. Standard deviations (SD) are presented in brackets. N.D.—not determined, 95% CI—95% confidence interval.
