# Supplementary material for: Application of a Novel Dissolution Medium with Lipids for In Vitro Simulation of the Postprandial Gastric Content
Source: Pharmaceutics. 2024 Aug 3;16(8):1040. doi: 10.3390/pharmaceutics16081040 (PMC11359312; doi:10.3390/pharmaceutics16081040)
Supplement: Supplementary file 1 [file pharmaceutics-16-01040-s001.zip › pharmaceutics-3128405-supplementary.pdf]

SUPPLEMENTARY MATERIALS

Application of a Novel Dissolution Medium with Lipids for In Vitro Simulation of the Postprandial Gastric Content

Tjaša Felicijan , Iva Rakoše , Manca Prislan , Igor Locatelli , Marija Bogataj *, and Jurij Trontelj

Department of Biopharmaceutics and Pharmacokinetics, faculty of Pharmacy, University of Ljubljana, Akerčeva cesta 7, 1000 Ljubljana, Slovenia;
tjasa.felicijan@ffa.uni-lj.si (T.F.); iva.rakose@gmail.com (I.R.);
manca.prislan@hotmail.com (M.P.); igor.locatelli@ffa.uni-lj.si (I.L.);
jurij.trontelj@ffa.uni-lj.si (J.T.)
* Correspondence: marija.bogataj@ffa.uni-lj.si

1. The composition of SMOFlipid®

100 mL of SMOFlipid® is composed of the following [45]:

- 6 g soybean oil
- 6 g medium-chain triglycerides
- 5 g olive oil
- 3 g fish oil
- 1.2 g egg phospholipids
- 2.5 g glycerin
- 16.3 to 22.5 mg all-rac-alpha-tocopherol
- 0.03 g sodium oleate
- water for injection
- sodium hydroxide for pH adjustment (pH 6 to 9)

The energy content of emulsion is 2000 kcal/L. The lipid content is 0.2 g/L, and the oils used in the emulsion consist of predominantly unsaturated fatty acids (oleic acid, linoleic acid, caprylic acid, palmitic acid, alpha-linolenic acid, eicosapentaenoic acid, docosahexaenoic acid.

45. SMOFlipid® (Lipid Injectable Emulsion), for Intravenous Use [Highlights of Prescribing Information]. Available online: https://www.freseniuskabinutrition.com/wp-content/uploads/2023/10/US-PH-SMOFlipid-FK-451418E-PI.pdf (accessed on 23 April 2023).

2. Assumptions used in calculations to determine the amount of CIN in the separate phases

The following assumptions were adopted to calculate the CIN concentrations in the lipid and aqueous phases from the responses measured by HPLC analyses of the separate phases. Complete separation of the emulsion could not be achieved by centrifugation, and the obtained lipid-rich phase was thus not composed solely of lipid but was a lipid-rich emulsion containing a noticeable proportion of the aqueous phase itself. After withdrawing the aliquot of the lower phase, the remaining sample volume in the microcentrifuge (300 µL) contained the upper lipid-rich phase and the residual of the aqueous phase, as it was not possible to withdraw the entire volume of the aqueous phase. Therefore, theoretical volumes of lipid and aqueous phases were used to calculate the CIN distribution between aqueous and lipid phases. The volume of the lipid phase in the medium was determined from the mass of the lipids in the medium considering the SMOFlipid® dilution with buffer and the density of soy oil (0.917 mg/L [64]), which is one of the components of the parenteral lipid emulsion. The remaining volume of the sample was thus assumed to be the theoretical volume of the aqueous phase.

Such theoretical volume was then used when calculating the amount of CIN in the aqueous phase from the analyte response in the sample's lower phase (procedure 2a in Figure 1 in the main text). It was thus hypothesized that after phase centrifugation, the lower phase did not contain any lipids and consisted of pure aqueous phase. The amount of CIN in the lipid phase was calculated from the analyte response in the remaining 300 µL of the sample after phase separation and withdrawal of 200 µL of the lower phase (procedure 2b in Figure 1 in the main text). Here, we assumed that the volume of the remaining sample was a sum of the volumes of the lipid phase and the remaining aqueous phase in the sample after withdrawing 200 µL of the lower phase. The residual volume of the aqueous phase in the upper lipid-rich phase of the sample was calculated by subtracting the volume of the withdrawn lower phase from the theoretical volume of the aqueous phase in the sample. The concentration of CIN in the aqueous phase was assumed to be equal in the lower pure aqueous phase and the aqueous part of the upper lipid-rich phase. Thus, by subtracting the mass of CIN found in the aqueous part of the analyzed sample containing the upper lipid-rich phase from the total determined mass of CIN in the upper phase sample, the amount of CIN in the lipid phase was calculated.

64. Merck. Soybean Oil. Available online: https://www.sigmaaldrich.com/SI/en/product/usp/1617500 (accessed on 23 April 2024).

3. HPLC method validation – analyte stability and method robustness

The analyte peak responses and corresponding calculated concentration of quality control (QC) standards to test method robustness are presented in Tables S1a-g. Validation was performed at three QC levels (QCL-2, QCM, and QCH, with concentrations around 35, 75, and 115 mg/L) in a medium at pH 3 and a 100 mg/L lipid concentration. The final method parameters and analyte responses, used as reference values for accuracy calculation, are presented in **bold**.

Table S1a: Testing the changes in the column temperature (± 5°C) on analyte response.

column temperature									
	**45°C**			40 °C			50 °C		
	**QCL-2**	**QCM**	**QCH**	QCL-2	QCM	QCH	QCL-2	QCM	QCH
peak area (mAUs)	**74.1**	**162.6**	**252.3**	74.2	161.9	252	74.6	162.1	253.1
c (mg/L) (calculated)	**35.8**	**77.8**	**120.4**	35.8	77.5	120.3	36.0	77.6	120.8
**accuracy (%)**				100.1	99.6	99.9	100.7	99.7	100.3

Table S1b: Testing the changes in the detector wavelength (± 2 nm) on analyte response.

detector wavelength									
	**251 nm**			249 nm			253 nm		
	**QCL-2**	**QCM**	**QCH**	QCL-2	QCM	QCH	QCL-2	QCM	QCH
peak area (mAUs)	**74.1**	**162.6**	**252.3**	73.8	159.7	248.1	74.1	161.8	250.9
c (mg/L) (calculated)	**35.8**	**77.8**	**120.4**	35.6	76.4	118.4	35.8	77.4	119.8
**accuracy (%)**				99.6	98.2	98.3	100.0	99.5	99.4

Table S1c: Testing the changes in mobile phase flow rate (± 0.15 mL/min) on analyte response. A ratio between the tested and reference mobile phase flow rate was used to calculate the expected concentration at the tested flow rates.

mobile phase flow rate									
	**1.5 mL/min**			1.35 mL/min			1.65 mL/min		
	**QCL-2**	**QCM**	**QCH**	QCL-2	QCM	QCH	QCL-2	QCM	QCH
peak area (mAUs)	**74.1**	**162.6**	**252.3**	82.0	178.3	277.7	67.8	148	228.3
c (mg/L) (calculated)	**35.8**	**77.8**	**120.4**	35.6	76.7	119.3	36.0	78.0	119.9
**accuracy (%)**				99.4	98.6	99.0	100.8	100.2	99.6

Table S1d: Testing the changes in the initial percentage of the mobile phase A (± 5%) on analyte response.

the percentage of mobile phase A at a final gradient									
	**65%**			60%			70%		
	**QCL-2**	**QCM**	**QCH**	QCL-2	QCM	QCH	QCL-2	QCM	QCH
peak area (mAUs)	**74.1**	**162.6**	**252.3**	74.1	161.6	251.5	74.0	161.5	251.9
c (mg/L) (calculated)	**35.8**	**77.8**	**120.4**	35.8	77.3	120.0	35.7	77.3	120.2
**accuracy (%)**				100.0	99.4	99.7	99.9	99.3	99.8

Table S1e: Testing the changes in mobile phase A pH value (± 0.3 units) on analyte response.

mobile phase A pH value									
	**2.52**			2.27			2.78		
	**QCL-2**	**QCM**	**QCH**	QCL-2	QCM	QCH	QCL-2	QCM	QCH
peak area (mAUs)	**74.8**	**162.9**	**252.2**	76.4	166.5	258.7	80.5	173.8	268.1
c (mg/L) (calculated)	**36.1**	**78.0**	**120.4**	36.9	79.7	123.5	38.8	83.1	127.9
**accuracy (%)**				102.1	102.2	102.6	107.4	106.6	106.3

Table S1f: Testing the changes in injection volume (± 1 µL) on analyte response. A ratio between tested and reference injection volumes was used to calculate the expected concentration at tested injection volumes.

injection volume									
	**5 µL**			4 µL			6 µL		
	**QCL-2**	**QCM**	**QCH**	QCL-2	QCM	QCH	QCL-2	QCM	QCH
peak area (mAUs)	**74.1**	**162.6**	**252.3**	59.2	129.5	200	89.2	194.6	302.9
c (mg/L) (calculated)	**35.8**	**77.8**	**120.4**	35.8	77.6	119.5	35.8	77.5	120.4
**accuracy (%)**				100.2	99.7	99.2	100.1	99.6	100.0

Table S1g: Automatic and manual integration of seven quality QC standards with nominal CIN concentrations between approximately 7 and 175 mg/L. Manual integration was performed individually by two analysts.

analyte peak area (mAUs)							
	QC-1	QC-2	QC-3	QC-4	QC-5	QC-6	QC-7
automatic	13.3	72.1	106.9	147.9	239.3	263.1	375.3
analyst 1	13.1	71.4	106.6	148.0	238.0	259.7	370.7
analyst 2	13.4	71.6	106.9	147.3	238.8	262.4	374.2
average	13.3	71.7	106.8	147.7	238.7	261.7	373.4
**precision (% RSD)**	0.8	0.4	0.1	0.2	0.2	0.5	0.5

The analyte stability before sample preparation was tested at a pH value of 3, and both lipid concentrations (DMB pH 3 + lipid (8.8 mg/mL) and DMB pH 3 + lipid (100 mg/mL)) at three time points: 0 h, 2 h, and 4 h. The CIN concentration in samples was around 70 mg/L. The results are presented in Tables S2a and S2b.

Table S2a: The analyte stability before sample preparation in medium DMB pH 3 + lipid (8.8 mg/mL). The results of two or three parallels are presented for all three time points. The concentrations obtained at 0 h time point are used as a reference for accuracy calculation at other time points. The precision is calculated as % RSD from all measurements. N.D.—not determined.

DMB pH 3 + lipid (8.8 mg/mL)					
peak area (mAUs)			c (mg/L) (calculated)		
0 h	2 h	4 h	0 h	2 h	4 h
149.2	154.0	156.6	71,5	73,7	75,0
149.5	155.4	156.0	71,6	74,4	74,7
N.D.	152.9	154.4	N.D.	73,2	73,9
average			71.5	73.8	74.5
**accuracy (%)**				103.1	104.2
**precision (% RSD)**			1.7		

Table S2b: The analyte stability before sample preparation in medium DMB pH 3 + lipid (100 mg/mL). The results of two or three parallels are presented for all time points. The concentrations obtained at 0 h time point are used as a reference for accuracy calculations at other time points. The precision is calculated as % RSD from all measurements. N.D.—not determined.

DMB pH 3 + lipid (100 mg/mL)

peak area (mAUs)			c (mg/L) (calculated)		
0 h	2 h	4 h	0 h	2 h	4 h
156.1	158.8	157.2	74.7	76.0	75.2
156.1	160.0	159.1	74.7	76.6	76.1
N.D.	160.4	158.4	N.D.	76.8	75.8
average			74.7	76.5	75.7
**accuracy (%)**				102.3	101.3
**precision (% RSD)**			1.0		

The analyte stability after sample preparation was tested for seven QC standards with concentrations from around 7 to 175 mg/L at pH 3 with a lipid concentration of 100 mg/mL (DMB pH 3 + lipid (100 mg/mL)) at two time points, 0 h and 24 h. The results are presented in Table S2c.

Table S2c: The analyte stability after sample preparation in medium DMB pH 3 + lipid (100 mg/mL). The results are presented for two time points.

DMB pH 3 + lipid (100 mg/mL)

		QC-1	QC-2	QC-3	QC-4	QC-5	QC-6	QC-7
**0 h**	analyte peak area (mAUs)	13.9	72.2	107.7	148.5	239.4	263.7	375.0
**24 h**		13.3	72.1	106.9	147.9	239.3	263.1	375.3
**0 h**	c (mg/L) (calculated)	6.5	34.9	51.7	71.1	114.3	125.8	178.7
**24 h**		6.3	34.8	51.3	70.8	114.3	125.6	178.9
**accuracy (%)**		96.0	99.9	99.2	99.6	100.0	99.8	100.1

4. Mass balance confirmation experiments

The measured concentration of CIN in the whole sample and the percentage of CIN in aqueous and lipid phases for all measured time points in mass balance experiments are presented in Tables S3a and S3b.

Table S3a: Mass balance results in media with a lipid concentration of 8.8 mg/mL at three pH values. The average determined concentration (mg/L) of CIN in the whole sample and the average percentage of CIN in the aqueous and lipid phases of three parallels at different time points after standard preparation (0 h, 4 h, 24 h) are presented. Standard deviations (SD) are presented in brackets. N.D.—not determined, 95% CI–95% confidence interval.

		**DMB pH 3 + lipid 8.8 mg/mL**			**DMB pH 5 + lipid 8.8 mg/mL**			**DMB pH 7 + lipid 8.8 mg/mL**		
	**t (h)**	QCL-2	QCM	QCH	QCL-2	QCM	QCH	QCL-2	QCM	QCH
**CIN conc. in the whole sample (mg/L) (SD)**	**0**	37.34 (0.88)	73.78 (0.58)	121.87 (1.32)	37.27 (1.11)	74.01 (0.25)	122.75 (0.23)	37.60 (0.53)	75.30 (0.36)	124.09 (0.30)
**% in aqueous phase (SD)**	**0**	60.07 (2.89)	71.16 (0.17)	73.16 (1.49)	15.13 (0.28)	15.31 (1.12)	14.77 (0.80)	5.06 (0.78)	15.36 (0.88)	4.48 (1.98)
**% in lipid phase (SD)**	**0**	38.73* (1.33)	27.22* (0.57)	25.57* (0.32)	84.39* (0.44)	82.12* (0.41)	83.51* (1.00)	93.85* (0.77)	78.91* (3.14)	91.97* (1.40)
**% in aqueous phase (SD)**	**4**	63.68 (0.23)	N.D.	74.69 (0.73)	20.60 (1.28)	N.D.	15.85 (1.16)	14.54 (3.15)	N.D.	5.45 (1.30)
**% in lipid phase (SD)**	**4**	34.58* (0.82)	N.D.	23.96* (0.58)	76.76* (1.62)	N.D.	82.87* (0.14)	84.42* (1.65)	N.D.	91.65* (1.42)
**% in aqueous phase (SD)**	**24**	59.51 (2.71)	N.D.	76.11 (0.19)	18.38 (2.29)	N.D.	14.55 (1.01)	4.79 (0.60)	N.D.	5.01 (1.06)
**% in lipid phase (SD)**	**24**	40.94* (0.68)	N.D.	25.41* (0.20)	79.42* (1.75)	N.D.	84.02* (0.76)	94.97* (1.56)	N.D.	92.66* (0.38)
**mean of % in lipid phase of values with * (95% CI) used in ANOVA test**		30.9 (27.9–34.0) n = 21			81.9 (80.6–83.1) n = 21			89.8 (87.2–92.4) n = 21		

Table S3b: Mass balance results in media with a lipid concentration of 100 mg/mL at three pH values. The av-erage determined concentration (mg/L) of CIN in the whole sample and the average percentage of CIN in the aqueous and lipid phases of three parallels at different time points after standard preparation (0 h, 4 h, and 24 h) are presented. Standard deviations (SD) are presented in brackets. N.D. — not determined, 95% CI—95% confidence interval.

		**DMB pH 3 + lipid 100 mg/mL**			**DMB pH 5 + lipid 100 mg/mL**			**DMB pH 7 + lipid 100 mg/mL**		
	**t (h)**	QCL-2	QCM	QCH	QCL-2	QCM	QCH	QCL-2	QCM	QCH
**CIN conc. in the whole sample (mg/L) (SD)**	**0**	38.53 (1.71)	76.45 (0.40)	119.67 (0.45)	38.12 (0.11)	73.60 (0.40)	117.81 (2.00)	36.79 (0.87)	74.44 (1.33)	120.81 (0.86)
**% in aqueous phase (SD)**	**0**	18.98 (0.34)	29.54 (0.02)	25.23 (0.33)	10.56 (0.19)	14.87 (0.6)	11.01 (0.33)	2.35 (0.41)	4.42 (0.47)	2.58 (0.55)
**% in lipid phase (SD)**	**0**	73.71* (1.64)	66.69* (0.53)	70.19* (0.65)	85.62* (1.27)	81.33* (1.22)	90.35* (0.21)	98.19* (1.45)	95.19* (0.82)	95.66* (0.58)
**% in aqueous phase (SD)**	**4**	34.34 (1.17)	N.D.	27.72 (0.11)	11.71 (0.78)	N.D.	15.12 (0.42)	7.01 (2.55)	N.D.	1.02 (0.36)
**% in lipid phase (SD)**	**4**	62.04* (1.32)	N.D.	68.53* (0.84)	85.92* (0.90)	N.D.	89.08* (0.78)	94.09* (0.14)	N.D.	95.77* (0.62)
**% in aqueous phase (SD)**	**24**	43.04 (0.65)	N.D.	27.38 (0.13)	13.61 (0.73)	N.D.	20.68 (0.47)	2.85 (0.37)	N.D.	3.35 (0.13)
**% in lipid phase (SD)**	**24**	53.72* (1.76)	N.D.	69.64* (0.28)	84.15* (0.36)	N.D.	83.67* (1.91)	97.90* (0.45)	N.D.	95.50* (1.18)
**mean of % in lipid phase of values with * (95% CI) used in ANOVA test**		66.4 (63.5–69.3) n = 21			85.7 (84.3–87.2) n = 21			96.0 (95.3–96.8) n = 21		
